# Deep sedation using propofol target-controlled infusion for gastrointestinal endoscopic procedures: a retrospective cohort study

**DOI:** 10.1186/s12871-020-01103-w

**Published:** 2020-08-10

**Authors:** María E. García Guzzo, María S. Fernandez, Delfina Sanchez Novas, Sandra S. Salgado, Sergio A. Terrasa, Gonzalo Domenech, Carlos A. Teijido

**Affiliations:** 1 Department of Anesthesiology, Hospital Italiano de Buenos Aires, Ciudad Autónoma de Buenos Aires, Presidente Teniente General Juan Domingo Perón 4190, 1199 Buenos Aires, Argentina; 2 Department of Research, Hospital Italiano de Buenos Aires, Ciudad Autónoma de Buenos Aires, Presidente Teniente General Juan Domingo Perón 4190, 1199 Buenos Aires, Argentina

**Keywords:** Propofol, Anaesthesiologists, Endoscopy, Gastrointestinal, Anaesthesia, intravenous, Deep sedation

## Abstract

**Background:**

Propofol sedation is effective for gastrointestinal endoscopic procedures, but its narrow therapeutic window highlights the importance of identifying an optimal administration technique regarding effectiveness and safety. This study aimed to determine the incidence of significant adverse events in adult patients scheduled for gastrointestinal endoscopy under anaesthetist-performed sedation using propofol target-controlled infusion and determine the existence of associations between these events and potentially related variables.

**Methods:**

This single-centre, retrospective cohort study took place in a tertiary referral university hospital. Medical records of 823 patients (age > 18 years, American Society of Anesthesiologists physical status classification scores I–III) who had undergone elective gastrointestinal endoscopy under propofol target-controlled infusion sedation during September 2018 were reviewed. Outcomes included hypoxia, hypotension, and bradycardia events, requirement of vasoactive drugs, unplanned tracheal intubation or supraglottic device insertion, and need for advanced cardiac life support.

**Results:**

The most frequently encountered adverse event was oxygen desaturation < 95% with an incidence of 22.35%. Vasoactive drug administration, hypotension, and oxygen desaturation < 90% followed, with incidences of 19.2, 12.64, and 9.92%, respectively. Only 0.5% of patients required advanced airway management. Multivariate analysis revealed an association between hypotension events, colonoscopic procedures, and propofol doses (odds ratio: 3.08, 95% confidence interval: 1.43 to 6.61; *P* = 0.004 and odds ratio: 1.14, 95% confidence interval: 1.00 to 1.29; *P* = 0.046). A strong dose-effect relationship was found between hypoxia and obesity; patients with body mass index ≥40 were nine times (odds ratio: 10.22, 95% confidence interval: 2.83 to 36.99) more likely to experience oxygen desaturation < 90% events.

**Conclusions:**

Propofol sedation using target-controlled infusion appears to be a safe and effective anaesthetic technique for gastrointestinal endoscopic procedures with acceptable rates of adverse events and could be more widely adopted in clinical practice.

## Background

Presently, gastrointestinal endoscopic procedures (GIEPs) are mostly performed under sedation. In a few patients, general anaesthesia remains necessary due to procedure invasiveness or patient characteristics [1, 2]. Benzodiazepines combined with opioids, usually referred to as traditional sedative agents, are widely used for sedation by anaesthesia care providers and gastroenterologists in the US and elsewhere [3]. However, randomised studies have evaluated pharmacological alternatives for procedural sedation and have shown that propofol (Baxter International Inc., Deerfield, IL), compared with the use of traditional agents, has a rapid onset of action, provides predictable sedation depth and recovery times, and improves patient satisfaction rates [4–6]. Nonetheless, and despite its beneficial pharmacokinetic profile, propofol has a narrow therapeutic window and no antidote, highlighting the fact that it should be administered by certified health professionals using precise administration techniques to avoid critical events [7].

Worldwide, the safety profiles of different models of propofol administration have been analysed, such as nurse- and gastroenterologist-performed sedation, patient-controlled and computer-assisted methods, and anaesthetist-managed propofol sedation using intermittent boluses [8–10]. Among the studied models, propofol administration using target-controlled infusion (TCI) systems is emerging as an attractive alternative for anaesthetist-managed sedation. The current propofol TCI systems are pre-programmed with the Marsh and Schnider pharmacokinetic models. The rate constants in the Marsh model are fixed, whereas compartment volumes and clearances are weight proportionally; the Schnider model has several fixed values, whereas others are adjusted according to total weight, lean body mass, and height. One major benefit of the Schnider model is that it adjusts the doses and infusion rates according to patient age [11]. Through the use of microprocessor-controlled infusion pumps, the infusion rate is dynamically titrated to achieve plasmatic or effect site ‘targeted’ concentrations [12]. Over the past 5 years at our institution most gastrointestinal endoscopies have been performed under propofol TCI sedation administered by anaesthetists. Although this technique has proven to be effective, few investigations have reported the incidence of unplanned cardiovascular and respiratory adverse events during anaesthetist-managed propofol TCI sedation for GIEPs [13]. Therefore, sufficient evidence for improved safety over other anaesthetic strategies is lacking.

The primary aim of this historical cohort study was to describe the incidence of significant adverse events (cardiovascular and respiratory) in adult patients scheduled for elective outpatient GIEPs under anaesthetist-performed propofol TCI sedation and nasal cannula oxygenation. The secondary aim was to investigate associations between hypotension and oxygen desaturation events and potentially related variables (procedure type and duration, opioid administration, and total propofol dose among others) through multivariate analysis.

## Methods

We conducted a single-centre retrospective cohort study in a tertiary referral university hospital. Electronic medical records of 823 adult patients were reviewed based on sample-size calculation. The included patients were ≥ 18 years old, had American Society of Anesthesiologists (ASA) physical status classification scores I–III, and underwent elective outpatient GIEPs managed by anaesthetists using propofol TCI sedation during September 2018. According to the recommendations of the Anesthesiology Department, all gastrointestinal endoscopies are performed using TCI propofol sedation, with the exception of patients requiering general anesthesia due to individual or procedural characteristics or presenting contraindications to propofol administration. Patients scheduled for endoscopic retrograde cholangiopancreatography, enteroscopy, and procedures performed under planned tracheal intubation were excluded from the study.

Ethical approval was provided by the Ethics Committee of the Hospital Italiano de Buenos Aires, Buenos Aires, Argentina (Chairperson: Dr. Augusto Pérez, Ethical Committee N° 3885) on September 24, 2018. The requirement for written informed consent was waived due to the retrospective nature of the study.

The patient variables recorded included sex, age, ASA physical status classification score, weight, height, and significant comorbidities (diabetes mellitus, arterial hypertension, dyslipidaemia, current/former tobacco smoking, coronary artery, cerebrovascular, and peripheral vascular diseases; congestive heart failure, chronic obstructive pulmonary disease, and obstructive sleep apnea). The procedures comprised diagnostic and therapeutic esophagogastroduodenoscopy (EGD), colonoscopy, or both. Procedure-related data recorded included anaesthetic strategy (propofol TCI sedation), total propofol dose (mg), use of opioid drugs (fentanyl), and total fentanyl dose (mcg); type of procedure (EGD, colonoscopy, or combined procedure), and procedure duration (minutes; including time for monitoring and nasal cannula placement, operating room (OR) checklist, and the procedure itself). Intra-procedural adverse events recorded were oxygen saturation (SaO_2_) < 90 and < 95%, arterial hypotension (systolic blood pressure < 90 mmHg), vasoactive drug administration, significant bradycardia (heart rate < 50 beats/minute or chronotropic drug requirement), unplanned supraglottic device (SGD) insertion or endotracheal intubation, and need for advanced cardiac life support (ACLS). Post-procedural data recorded consisted of oxygen saturation < 90 and < 95% events, arterial hypotension, nausea and/or vomiting episodes or administration of antiemetic drugs, time to post anaesthetic care unit (PACU) discharge (minutes), unplanned post procedural hospitalisation, and need for ACLS.

The main data sources comprised electronic medical records, including procedure subsections with attached anaesthetic and post-anaesthetic charts (PDF documents). Anaesthetic charts are automatically generated during procedures by electronic capture of real-time vital-sign measurements from multiparameter monitors and graphically displayed. Drug administration and comments on adverse events are manually entered by anaesthesiologists. Upon case completion, a PDF document is generated and available for review in the procedure subsection. During the post-procedure period at the PACU, vital-sign measurements and drug administration are entered by PACU nurses, and a post-anaesthetic chart is generated upon discharge.

All patients scheduled for colonoscopy received bowel cleansing preparation (Pico-Sulphate - Picoprep® or Polyethylene glycol - Barex®/Barex Kit®) before the procedure according to standardised hospital protocols. Vital signs were monitored (pulse oximeter, noninvasive blood pressure cuff, and five lead electrocardiography) once inside the operating room and after intravenous cannulation. Supplementary oxygen was delivered through a nasal cannula, and propofol was administered using TCI systems at titrated doses aiming for a deep level of sedation [14]. The TCI system included a Perfusor® Space pump (B. Braun Melsungen AG, Melsungen, Germany) controlled by a microprocessor system. After introducing patient data, the Schnider or Marsh pharmacokinetic models and initial target concentrations were selected by the anaesthesiologist. Throughout the procedures, automatic adjustments to the infusion rate were generated by perfusor pumps based on the predicted plasma or tissue levels of propofol and the target concentrations set by the operator, guided by clinical judgement.

All patients were transferred to the PACU immediately after completion of the procedure and were monitored for at least 30 min before discharge.

Our primary outcome was the incidence of adverse cardiovascular and/or respiratory events among patients undergoing GIEP under propofol TCI sedation. The secondary outcome was the existence of associations between hypotension and oxygen desaturation events and potentially related variables.

### Sample size calculation

Regarding the primary aim, we calculated a sample size of 823 patients based on an estimated incidence of 14% for SaO_2_ < 95% events and aiming for a CI with a hemi amplitude of 2.5% [13]. An incidence of 5% was estimated for SaO_2_ < 90% events in this population, predicting approximately 41 of these events among 823 patients. This allowed to build a multivariate model through the logistic regression technique including approximately four potentially related variables.

### Statistical analysis

Descriptive analyses were performed using the mean ± SD for continuous variables and numbers (proportions) for categorical variables. Qualitative variables derived from each group were compared using the χ^2^ test or Fisher’s exact test in cases involving low expected counts. Student’s t-test was used to analyse normally distributed quantitative data, while the nonparametric Wilcoxon rank-sum test was used to analyse non-normally distributed quantitative data. All statistical analyses were conducted using STATA.13 (StataCorp, College Station, TX).

We conducted two multivariate logistic regression analyses to investigate which variables would be associated with the development of SaO_2_ < 90% and hypotension events during propofol TCI sedation for GIEPs. For both models, variables were included based on significant associations (*P* < 0.1) with the event in the bivariate analysis. The final multivariate model for SaO_2_ < 90% included age, ASA physical status classification score, obesity, EGD procedure, propofol doses, and fentanyl administration; for hypotension events the model included age, ASA physical status classification score, history of arterial hypertension, obesity, colonoscopic procedures, propofol doses, and fentanyl administration.

Finally, we performed linear regression to examine whether propofol doses would decrease when administered together with fentanyl, adjusting for potentially confounding factors such as age, ASA classification status, and body mass index (BMI).

## Results

Medical records of 823 patients who underwent elective outpatient GIEPs under propofol TCI sedation were reviewed. Patients were aged 59.2 ± 14.1 years, 85% had ASA physical status classification scores I–II, and the mean BMI was 27.5 ± 4.8. The most frequently detected comorbidities were arterial hypertension (40.5%) and dyslipidaemia (34.5%) (Table 1)*.*

**Table 1:** Baseline characteristics

	TCI ***n*** = 823
Age, (years)	59.2 ± 14.1
Sex, (male)	352 (42.8%)
Weight. kg	74.6 ± 15.8
BMI (kg m^2–1^)	27.5 ± 4.8
ASA physical status, n (%)	
I	130 (15.8%)
II	567 (68.9%)
III	126 (15.3%)
Diabetes, n (%)	70 (8.5%)
Dyslipidaemia, n (%)	280 (34.5%)
Hypertension, n (%)	333 (40.5%)
Tobacco smoking, n (%)	119 (14.5%)
Coronary artery disease, n (%)	23 (2.8%)
Previous cerebrovascular disease, n (%)	12 (1.5%)

Results are presented as mean ± SD and number (proportion)

*BMI* body mass index, *ASA* American Society of Anesthesiologists

The most commonly performed procedure was colonoscopy alone (48.36%), followed by combined (EGD and colonoscopy) procedures (37.47%) (Table 2). The mean propofol dose was 291.2 ± 124.6 mg. Fentanyl was administered by 86.88% of anaesthetists combined with propofol at a mean dose of 0.77 ± 0.25 mcg kg^− 1^. The global mean procedure duration was 25.07 ± 11.43 min (Table 2).

**Table 2:** Procedure characteristics

Characteristic	EGD alone *n* = 166	Colonoscopy alone *n* = 398	EGD and colonoscopy *n* = 259	Total ***n*** = 823
**Duration of procedure**				
Global mean duration (minutes)	17.41 ± 10.25	24.15 ± 10.18	31.39 ± 10.51	25.07 ± 11.43
Post-procedure discharge time	41.26 ± 9.11	42.84 ± 10.73	44.79 ± 16.29	43.14 ± 12.54
**Medications**				
Propofol (mg)	229.31 ± 118.99	282.83 ± 112.66	343.73 ± 124.73	291 ± 124.6
Propofol (mg kg-1)	3.17 ± 1.62	3.82 ± 1.55	4.70 ± 1.56	3.97 ± 1.66
Fentanyl	146 (87.95)	329 (82.66)	240 (92.66)	715 (86.88)
Fentanyl (mcg)	54.11 ± 13.14	56.05 ± 16.84	56.14 ± 22.08	55.68 ± 18.14
Fentanyl (mcg kg^−1^)	0.75 ± 0.20	0.77 ± 0.25	0.78 ± 0.28	0.77 ± 0.25

Results are presented as number (proportion) and mean ± SD

*EGD* esophagogastroduodenoscopy

The most frequently encountered adverse event was SaO_2_ < 95%, with an incidence of 22.36% (184/823). Vasoactive drug administration and arterial hypotension followed, with incidences of 19.2% (158/823) and 12.64% (104/823), respectively. The incidence of SaO_2_ < 90% was 6.92% (57/823), while the incidence of bradycardia or chronotropic drug requirement was 4.73% (39/823). Only 0.5% of patients (4/823) required advanced airway management (unplanned SGD insertion or orotracheal intubation). No patients required ACLS or died during the procedure (Table 3). All patients were transferred to the PACU immediately after completion of the procedure and received home discharge at a mean post-procedure time of 43.1 ± 12.5 min.

**Table 3:** Intra-operative and post-operative outcomes

	EGD alone *n* = 166	Colonoscopy alone *n* = 398	EGD and colonoscopy *n* = 259	Total ***n*** = 823 (%) [95% CI]
**Intra-operative Outcomes**				
Hypoxia (SaO2 < 95%)	39 (23.49)	81 (20.35)	64 (24.71)	184 (22.36) [19.55 to 25.36]
Hypoxia (SaO2 < 90%)	15 (9.04)	25 (6.28)	17 (6.56)	57 (6.92) [5.29 to 8.88]
Hypotension (SBP < 90 mmHg)	8 (4.82)	71 (17.84)	25 (9.65)	104 (12.64) [10.44 to 15.10]
Administration of vasoactive drugs	19 (11.45)	93 (23.37)	46 (17.76)	158 (19.20) [16.56 to 22.06]
Bradycardia (HR < 50 beats per minute) or atropine administration	4 (2.41)	16 (4.02)	19 (7.34)	39 (4.74) [3.39 to 6.42]
Unplanned SGD insertion or tracheal intubation	2 (1.20)	2 (0.5)	0	4 (0.5) [0.13 to 1.24]
Need for ACLS	0	0	0	0 (0) [0 to 0.45]
**Post-operative outcomes**				
Hypoxia (SaO2 < 95%)	0	1 (0.25)	0	1 (0.12) [0 to 0.6]
Hypoxia (SaO2 < 90%)	0	0	0	0 (0) [0 to 0.45]
Hypotension (SBP < 90 mmHg)	8 (4.82)	24 (6.04)	6 (2.32)	38 (4.6) [3.29 to 6.28]
PONV	2 (1.20)	2 (0.5)	1 (0.39)	5 (0.6) [0.20 to 1.41]
Unplanned post procedural hospitalisation	0	0	0	0 (0) [0 to 0.45]

Results are presented as number (proportion), 95% CI

*EGD* esophagogastroduodenoscopy, *CI* confidence interval, *SaO*_*2*_ oxygen saturation, *SBP* systolic blood pressure, *HR* heart rate, *SGD* supraglottic device insertion, *ACLS* advanced cardiac life support, *PONV* post-operative nausea and/or vomiting

During the post-anaesthetic period, the most frequent adverse event was arterial hypotension with an incidence of 4.6% (38/823), followed by SaO_2_ < 95% events with an incidence of 0.12% (1/823). No patients presented events of SaO_2_ < 90%. The incidence of nausea/vomiting episodes was 0.6% (5/823). No patients required advanced airway management or ACLS during this period (Table 3).

Multivariate analysis revealed a statistically significant association between obesity (BMI > 30) and the incidence of SaO_2_ < 90% events (Table 4). This association depicted a strong dose–effect relationship; taking non-obese patients as a reference, the risk of those with BMI 30–35 (grade 1 obesity) was almost double, with an OR of 1,68 (CI 95% 0.84 to 3.32), while patients with BMI 35–40 (grade 2, severe obesity) and BMI ≥40 (grade 3, morbid obesity) were three times (OR 2.85, CI 95% 1.09 to 7.46) and nine times (OR 10.22, CI 95% 2.83 to 36.99) more likely to experience SaO_2_ < 90% events, respectively.

**Table 4:** Multivariable logistic regression analysis for the association between oxygen desaturation (< 90%) and potentially related factors

	OR	95% CI	***P***
**Grade 1 obesity**	1.68	0.85 to 3.33	0.14
**Grade 2 obesity**	2.86	1.09 to 7.46	0.03
**Grade 3 obesity**	10.23	2.83 to 36.99	**< 0.001**
**Age**	1	0.98 to 1.02	0.99
**ASA physical status II**	1.88	0.60 to 5.82	0.28
**ASA physical status III**	2.14	0.56 to 8.20	0.26
**EGD**	1.17	0.66 to 2.06	0.58
**Received Fentanyl**	0.91	0.40 to 2.07	0.80
**Propofol dose mg kg**^**−1**^	1.02	0.85 to 1.22	0.86

*OR* odds ratio, *CI* confidence interval, *ASA* American Society of Anesthesiologists, *EGD* esophagogastroduodenoscopy

For arterial hypotension events, multivariate analysis showed a statistically significant association between these events and both colonoscopic procedures and propofol doses (OR 3.08, 95% CI 1.43–6.61; *P* = 0.004 and OR 1.14, 95% CI 1.00–1.29; *P* = 0.046, respectively) (Table 5). After adjustment for potential confounders, a linear regression analysis showed a non-significant trend towards a reduction of 0.53 mg.kg- (95% CI − 0.86-0.2) in total propofol doses by concomitant fentanyl administration.

**Table 5:** Multivariable logistic regression analysis for the association between hypotension (< 90 mmHg) and potentially related factors

	OR	95% CI	***P***
**Colonoscopy**	3.08	1.43 to 6.61	0.004
**Propofol dose mg kg**^**−1**^	1.14	1.00 to 1.29	0.046
**Age**	0.99	0.97 to 1.01	0.24
**ASA physical status II**	1.85	0.95 to 3.59	0.07
**ASA physical status III**	1.18	0.46 to 3.02	0.73
**Hypertension**	0.77	0.46 to 1.27	0.305
**Received Fentanyl**	1.22	0.63 to 2.35	0.55

*OR* odds ratio, *CI* confidence interval, *ASA* American Society of Anesthesiologists

## Discussion

Few evidence-based studies have examined the safety outcomes of low-risk patients undergoing propofol TCI sedation for ambulatory GIEPs. Approximately 20,000 GIEPs are performed at our institution per year under sedation managed by anaesthetists, in accordance with institutional regulations. A safe anaesthetic technique with rapid turnover and discharge times is essential for this high-volume practice to remain efficient.

The study of our population demographics revealed that most patients scheduled for elective procedures on an outpatient basis had a low-risk profile (85% had ASA physical status classification scores I–II). Invasive diagnostic and therapeutic procedures were excluded from the investigation. The largest proportion of interventions consisted of colonoscopies alone, followed by colonoscopies combined with EGD; hence, we can state that this study involved a low-risk population undergoing low-risk procedures.

Regarding the administration of hypnotic drugs, Leslie et al. reported a mean total propofol dose of 200 mg in a study including more than 2000 patients undergoing GIEPs [9], which is lower than the mean total propofol dose administered in the current investigation (291.2 mg). Although a smaller proportion of patients in the study by Leslie et al. received propofol in combination with opioids (fentanyl or alfentanyl), the weight-adjusted doses of fentanyl were similar in both studies (0.78 vs. 0.77 mcg.kg^−^). In addition, 37% of patients in the aforementioned study also received 2 mg of midazolam during anaesthesia induction, which has been proven to reduce propofol requirements [15, 16]. Therefore, the lower administered doses of propofol in the study by Leslie et al. could probably be attributed to the addition of midazolam, and not opioids, to the anaesthetic regimen. Despite the fact that our patients received larger doses of propofol, this was not associated with an increased incidence of arterial hypotension events, which was reported to be up to 12% in both studies [9].

When comparing our results with those from a study conducted by Chang et al. [13], the administered propofol doses in our practice were higher, although dispensed using TCI in both investigations. Patients in the study by Chang et al. received propofol as well as 2–2.5 mg midazolam and a mean alfentanil dose of 493 ng. Again, combination of propofol TCI with benzodiazepines may account for the reduction in propofol requirements. 

The procedure duration recorded in the present study (median time 25.07 ± 11 min) was similar to that reported by Leslie et al. However, we found differences in discharge times from the PACU; our mean time to hospital discharge was 42 min, IQR [37 to 48] compared with 60 min, IQR [33 to 82] in the previous study. Although it is difficult to compare the results without knowledge of the discharge criteria used in other studies, extended stay in the PACU may be related to the use of benzodiazepines. These drugs appear to prolong recovery time at the expense of similar hypotension rates [17].

Arterial hypotension was one of the most frequently encountered adverse events, with an incidence of 12,64%. The administration of vasoactive drugs (19.6%) was more frequent than the occurrence of arterial hypotension, probably related to the fact that many anaesthesiologists selected to administer them pre-emptively to avoid hypotension events. Although these incidences may appear high, we did not encounter major cardiovascular complications and none of the patients included in our study required ACLS or died during the perioperative period.

Through multivariate analysis, colonoscopic procedures and higher propofol doses were found to be associated with a higher incidence of arterial hypotension events. As reported in the literature, bowel cleansing with sodium phosphate relates to significant orthostatic hypotension and increases in heart rate, probably due to intravascular volume contraction [18, 19]. This scenario can lead to an increased propensity to arterial hypotension events when combined with administration of propofol for deep sedation.

With respect to respiratory adverse events, SaO_2_ < 95% was the most frequently encountered event (22.3%); this drop in pulse oximetry likely represents upper airway obstructions with no significant clinical impact. Events of SaO_2_ < 90% were encountered less often with an incidence of 6.9%. Only four (0.5%) patients required unplanned orotracheal intubation, suggesting that most SaO_2_ < 95 and < 90% events were resolved by non-invasive manoeuvres to unclog the airway including chin lift, jaw thrust, or insertion of oral/nasal cannulas. Considering the fact that the present study involved elective gastrointestinal procedures in relatively low-risk patients, the rates of significant hypoxia (SpO2 < 90% events) of 6.92% and unplanned orotracheal intubation of 0.5% were non-trivial. The authors believe that certain quality improvements could be incorporated to clinical practice, such as electroencephalographic monitoring and capnography tracing, which may allow for further analyses concerning the appropriate anaesthetic depth, recommended TCI target concentrations, and oxygenation/respiratory adequacy.

Multivariate analysis revealed a significant association between SaO_2_ < 90% events and obesity. Morbid obesity showed a 10.22 OR for these events, probably suggesting the need for alternate airway management and/or oxygen supplementation strategies under sedation for these patients. The use of a high-flow nasal cannula or continuous positive airway pressure via nasal mask (SuperN2va) have been proposed to reduce oxygen desaturation events for spontaneously breathing obese patients [20, 21].

Most of the limitations of this study are related to information bias due to its retrospective nature. Hemodynamic variables were automatically captured from multiparameter monitors and graphically displayed. Although vasoactive drug administration data were obtained from these anaesthetic charts, the exact administration time registered by anaesthetists may not have been accurate; hence, it is difficult to determine whether these interventions were therapeutic or pre-emptive. Moreover, even when the total propofol doses were accurately documented due to institutional regulations for drug control, the TCI models (Marsch vs Schnider) and target concentrations selected for each patient throughout the procedure were not recorded. We did not encounter major events during the course of the procedures or the immediate post-operative period, and all patients were discharged from the hospital on the day of the procedure. Therefore, it is difficult to evaluate whether intraprocedural hypotension events had any long-term, directly related, cardiovascular or neurovascular consequences. Regarding oxygen desaturation, the use of non-invasive manoeuvres destined to increase oxygen saturation in patients registering SpO_2_ values under 95% is not regularly recorded in the anaesthetic chart.

## Conclusions

Patients undergoing GIEPs under propofol TCI sedation combined with fentanyl were not exempted from experiencing cardiovascular or respiratory unplanned events, specifically grade II and III obese patients who showed a significant dose-effect relationship with SpO2 < 90% events. Nonetheless, it appears to be an effective sedative strategy for GIEPs with the benefits of acceptable rates of unplanned adverse events, short discharge times, and rapid patient turnover. Further prospective studies should be conducted to accurately assess patient comfort and quality of sedation for this type of procedure.

## Data Availability

The datasets used and/or analysed during the current study available from the corresponding author on reasonable request.

## Abbreviations

ACLS: Advanced cardiac life support
ASA: American Society of Anesthesiologists
BMI: Body mass index
EGD: Esophagogastroduodenoscopy
ESA: European Society of Anesthesiology
GIEP: Gastrointestinal endoscopic procedure
HR: Heart rate
OR: Operating room
PACU: Post anaesthetic care unit
PONV: Post-operative nausea and/or vomiting
SBP: Systolic blood pressure
SGD: Supraglottic device
TCI: Target-controlled infusion
